# Factor XII in PMM2-CDG patients: role of N-glycosylation in the secretion and function of the first element of the contact pathway

**DOI:** 10.1186/s13023-020-01564-9

**Published:** 2020-10-09

**Authors:** Raquel López-Gálvez, María Eugenia de la Morena-Barrio, Alberto López-Lera, Monika Pathak, Antonia Miñano, Mercedes Serrano, Delphine Borgel, Vanessa Roldán, Vicente Vicente, Jonas Emsley, Javier Corral

**Affiliations:** 1 Servicio de Hematología y Oncología Médica, Hospital Universitario Morales Meseguer, Centro Regional de Hemodonación, Universidad de Murcia, IMIB-Arrixaca, CIBERER, Ronda de Garay S/N, 30003 Murcia, Spain; 2 Instituto de Investigación Sanitaria del Hospital La Paz (IdiPaz), Madrid, Spain; 3 Centre for Biomedical Network Research On Rare Diseases (CIBERER) U-754, Hospital Universitario La Paz, Madrid, Spain; 4 Centre for Biomolecular Sciences, School of Pharmacy, University of Nottingham, Nottingham, NG7 2RD England; 5 Departamento de Neurología Pediátrica, Departamento de Bioquímica Clínica, Instituto de Investigación Pediátrica-Hospital Sant Joan de Déu, CIBERER U-703, Barcelona, Spain; 6 Laboratoire D’Hématologie, AP-HP, Hôpital Necker-Enfants Malades, Paris, France; 7 UMR-S1176, Université Paris-Saclay, INSERM, 94276 Le Kremlin-Bicêtre, France

**Keywords:** Factor XII, Congenital disorders of glycosylation, N-glycosylation, Angioedema, Hemostasis

## Abstract

**Background:**

Congenital disorders of glycosylation (CDG) are rare diseases with impaired glycosylation and multiorgan disfunction, including hemostatic and inflammatory disorders. Factor XII (FXII), the first element of the contact phase, has an emerging role in hemostasia and inflammation. FXII deficiency protects against thrombosis and the p.Thr309Lys variant is involved in hereditary angioedema through the hyperreactivity caused by the associated defective O-glycosylation. We studied FXII in CDG aiming to supply further information of the glycosylation of this molecule, and its functional and clinical effects. Plasma FXII from 46 PMM2-CDG patients was evaluated by coagulometric and by Western Blot in basal conditions, treated with N-glycosydase F or activated by silica or dextran sulfate. A recombinant FXII expression model was used to validate the secretion and glycosylation of wild-type and variants targeting the two described FXII N-glycosylation sites (p.Asn230Lys; p.Asn414Lys) as well as the p.Thr309Lys variant.

**Results:**

PMM2-CDG patients had normal FXII levels (117%) but high proportions of a form lacking N-glycosylation at Asn414. Recombinant FXII p.Asn230Lys, and p.Asn230Lys&p.Asn414Lys had impaired secretion and increased intracellular retention compared to wild-type, p.Thr309Lys and p.Asn414Lys variants. The hypoglycosylated form of PMM2-CDG activated similarly than FXII fully glycosylated. Accordingly, no PMM2-CDG had angioedema. FXII levels did not associate to vascular events, but hypoglycosylated FXII, like hypoglycosylated transferrin, antithrombin and FXI levels did it.

**Conclusions:**

N-glycosylation at Asn230 is essential for FXII secretion. PMM2-CDG have high levels of FXII lacking N-glycosylation at Asn414, but this glycoform displays similar activation than fully glycosylated, explaining the absence of angioedema in CDG.

## Introduction

There is a renaissance in studying the role of factor XII (FXII) in pathways leading to thrombosis and inflammation. No bleeding is associated with FXII deficiency observed in different animal models (mice and cats) [1, 2], and in FXII-deficient patients [3], which has dramatically changed by the observed antithrombotic protection caused by the severe reduction of FXII levels in mice [4–6], rats [7], rabbit [8, 9], or baboons [10]. Additionally, different FXII variants affecting the proline-rich domain, particularly the recurrent Thr309Lys mutation, have been recently involved in a type of life-threatening inherited swelling disorder, hereditary angioedema (HAE) [11–14].

FXII is a precursor of a serine protease that initiates the procoagulant and proinflammatory protease cascades. It becomes autoactivated following binding to negatively charged either artificial or biological surfaces. Kallikrein is also able to convert FXII into an active protease. Activated FXII (FXIIa) may promote both the activation of its procoagulant substrate factor XI (FXI), and the release of the proinflammatory mediator bradikinin (BK) by kallikrein-mediated cleavage of high molecular weight kininogen [15].

Glycosylation is a common post-translational modification (PTM) that plays key roles in folding, secretion, stability and function of multiple glycoproteins [16]. This PTM may be particularly relevant for FXII. Actually, the impaired O-glycosylation caused by the p.Thr309Lys mutation has been involved in the increased sensibility of this variant to become activated [17].

Type 1 congenital disorders of glycosylation (CDG) is a wide and heterogeneous group of rare autosomal recessive disorders that have impaired the N-glycosylation due to defects affecting one out of up to 25 different enzymes involved in the generation of the N-glycan precursor [18]. The most common, although still very rare CDG, is PMM2-CDG, caused by mutations in *PMM2*, which encodes the phosphomannomutase 2, an enzyme involved in the transformation of mannose-6-phosphate into mannose-1-phosphate, one of the first steps of N-glycosylation [19]. Thus, PMM2-CDG patients have increased levels of hypoglycosylated forms of multiple N-glycoproteins, particularly of hepatic origin. Actually, the identification of hypoglycosylated forms of transferrin is one gold standard in the diagnosis of PMM2-CDG [20].

**Fig. 1:**
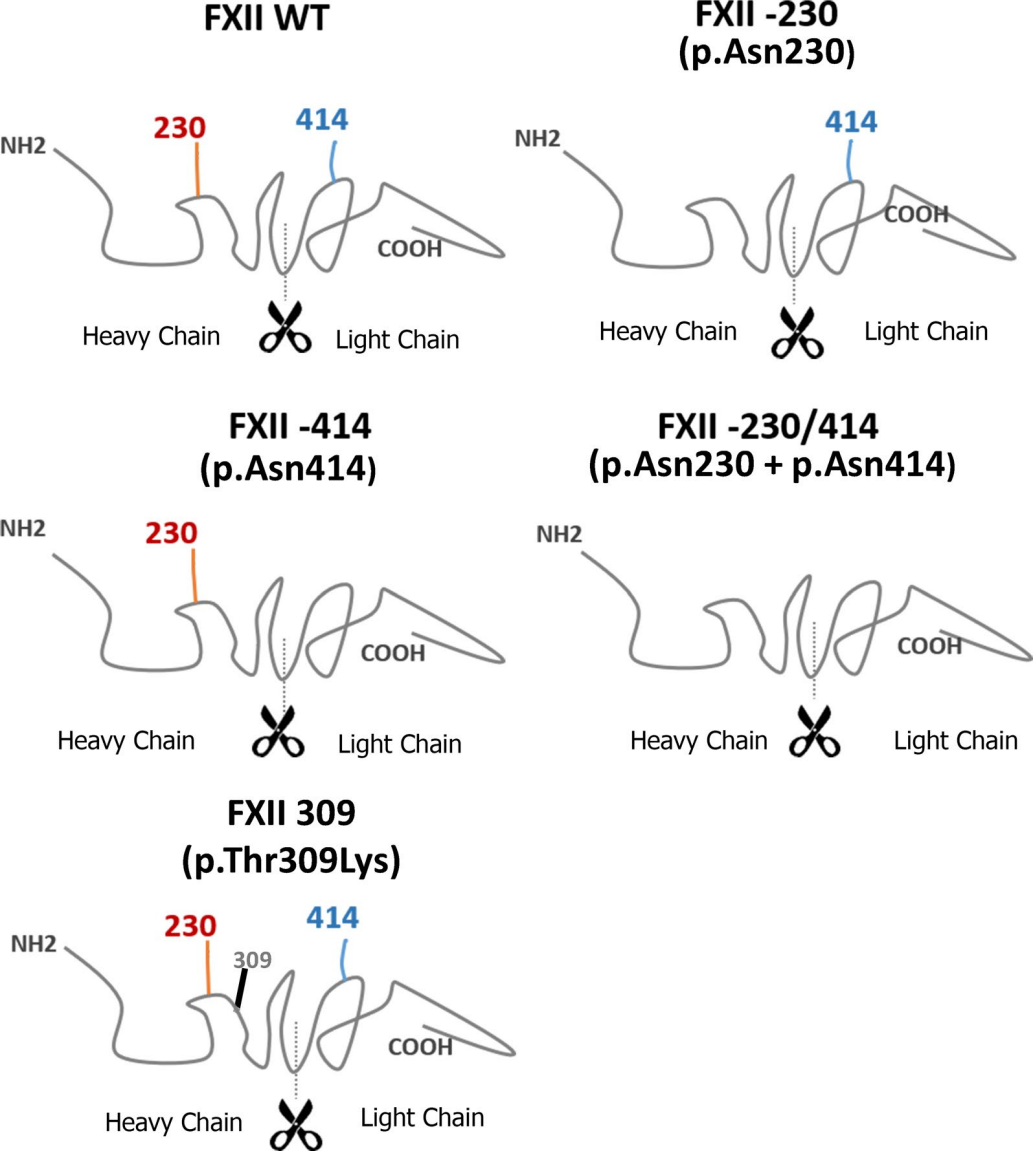
N-glycan sequons identified in FXII and FXII variants generated in the recombinant model. N-glycosylation positions are located in positions 230 (heavy chain) and 414 (light chain). The cleavage site of the heavy chain generated by activation of FXII is shown. As a control, the mutation involved in FXII-HAE p.Thr309Lys, also located in the heavy chain, was also generated

We proposed that the analysis of FXII, a hepatic 80 kDa glycoprotein containing 2 N-glycosylation sites (one in the heavy chain -Asn230- and the other one in the light chain -Asn414- Fig. 1), in a large cohort of PMM2-CDG patients together with a recombinant model deleting the two N-glycosylation signals might help to understand the role of N-glycosylation in FXII, and the clinical impact of an impaired N-glycosylation of this molecule in PMM2-CDG patients.

## Methods

### Patients

Forty-six patients with confirmed PMM2-CDG were retrospectively recruited from 6 hospitals [Spain (2), France (2), Portugal (1), and Belgium (1)]. The clinical characteristics of the main cohort of these patients are described elsewhere [21]. Glycoforms of transferrin; antithrombin activity and FXI levels were quantified by HPLC, a chromogenic assay, and a coagulometric method, as described previously [22].

### Genetic analysis

The PMM2-CDG variants were identified by NGS methods. Moreover, the *rs1801020* polymorphism affecting the Kozak sequence of *F12* that significantly modulates the levels of FXII in plasma [23], was genotyped by a TaqMan assay (C___1989313_20) (ThermoFisher).

### Blood collection

Blood samples were collected in citrate-anticoagulated tubes (1:10) and processed immediately. The tubes were centrifuged at 2500 rpm for 20 min at room temperature and the separated plasma was aliquoted and stored at − 70 °C until analysis.

Genomic DNA was purified by the salting out following Puregen Blood Core Kit (Quiagen) procedure and stored at − 20 °C.

### Ethical issues

The study was performed in accordance with the principles of the Declaration of Helsinki and approved by the ethics committee of Universitario Reina Sofía (8/2013). Written informed consent from patients and healthy subjects was provided.

### FXII evaluation in plasma

#### Functional and antigenic evaluation of FXII

The coagulant levels of FXII in plasma (FXII: C) of PMM2-CDG patients were measured using a coagulometric method in an automatic analyzer ACL TOP 700 after activating the contact pathway with micronized silica (SynthASil; 0020006800, HemosIL®, Instrumentation Laboratory) and diluting the plasma of patients in a factor XII deficient plasma (0020011210, HemosIL®, Instrumentation Laboratory). Results were shown as percentage of a reference plasma (Instrumentation Laboratory).

Antigen levels and forms of plasma FXII in PMM2-CDG patients were studied by Western Blot using denaturing polyacrilamide electrophoresis (8% PAGE) in the presence and absence of reducing agents (DTT). The blotting was performed by using a goat anti-human FXII polyclonal antibody (GAFXII-AP, Enzyme Research Laboratories), which only recognizes the heavy chain, followed by the goat anti-IgG secondary antibody coupled to peroxidase (A-9452-1VL, Sigma-Aldrich) as secondary antibody. Peroxidase was detected with a chemiluminescence system (ECL™, GE Healthcare) in an Image Quant LAS4000mini equipment (ExonBiotec, GE Healthcare).

Glycosylation of other hepatic proteins, antithrombin, α1-antitrypsin, FXI and transferrin was evaluated by Western Blot, Q-TOF, or HPLC as described elsewhere [22].

Densitometric analysis of glycoform bands was performed using Image J [24].

#### Analysis of FXII N-glycosylation

To characterize the N-glycan content of FXII, plasma of patients and controls (2 μL) were denatured for 5 min at 100 °C in 150 mM sodium phosphate buffer, pH 7.4 and 10% NP-40. Subsequently, samples were treated with 2 U of N-glycosidase F (PNGase-F) (Roche Diagnostics GmbH,) at 37 °C for 15 h and analyzed by Western Blotting as described before.

#### Activation of plasma FXII

Two different agents were used to activate plasma FXII: i) micronized silica (SynthAsil 0,020,006,800, HemosIL®, Instrumentation Laboratory) at different dilutions (stock solution and 1/10, 1/20 and 1/50 dilutions), for 10 min at 37 °C; ii) dextran sulfate (DXS) (Pharmacia) from 0.2 μg/mL to 0.001 μg/mL, for 30 min at 37 °C. The activation of FXII was then evaluated by Western Blotting, as described before.

### Recombinant FXII

We used the hFXII-full plasmid, which contains the cDNA sequence of human the *F12* gene. Four mutants were generated by site directed mutagenesis (Quik Change II Site-Directed Mutagenesis kit, Agilent technologies). The two N-glycosylation sites of FXII were abolished by changing the Asparagine (Asn) AAC codon, at position 230 or 414, to the Lysine (Lys) AAA codon. The two single and the double mutants were generated: p.Asn230Lys, p.Asn414Lys and p.Asn230Lys&Asn414Lys. As an additional control, the p.Thr309Lys mutation was also generated (Fig. 1).

The oligonucleotides used for site directed mutagenesis are shown in Table 1. All the mutants generated were verified by Sanger sequencing.

**Table 1:** Oligonucleotides used to carry out the site directed mutagenesis in the human *F12* cDNA that remove the two N-glycosylation sites

Mutation	Oligonucleotide sequence (5′–3′)
Asn230Lys	Forward: CACCTACCGGAAAGTGACTGCCGAG
	Reverse: CTCGGCAGTCACTTTCCGGTAGGTG
Asn414Lys	Forward: CAGGAACGCCGTAAACACAGCTGTGAGC
	Reverse: GCTCACAGCTGTGTTTACGGCGTTCCG
Thr309Lys	Forward: GAAGCCTCAGCCCAAGACCCGGACCCCGC
	Reverse: GCGGGGTCCGGGTCTTGGGCTGAGGTTC

Drosophila *Schneider 2* (S2) cells were cultured in complete Drosophila Schneider medium supplemented with 10% fetal bovine serum at 28 °C (Gibco; ThermoFisher Scientific). Transfection of these cells with the appropriate plasmids (30 ng/μl) in HEPES buffered saline solution (50 mM HEPES, 1.5 mM Na2HPO4, 280 mMNaCl, pH 7.1) was carried out with the calcium phosphate method following the manufacturer's protocol (Calcium Phosphate Transfection Kit; ThermoFisher Scientific). The cells were cultured for an additional 48 h before the addition of puromycin, which allows the selection of cells expressing the plasmid. The stable cells were then adapted to insect culture medium Serum-free Express Five (SFM) (Invitrogen). Subsequently, the SFM medium containing the secreted proteins was collected due to the induction of the cells with CuSO_4_, and the cell pellet was removed by centrifugation.

### Statistical analysis

Statistical analysis were performed using the SPSS software (version 15.0). Correlation studies between the degree of hypoglycosylation and asialotransferrin or antithrombin levels were analysed using Pearson test. Mean comparisons were performed with T-Student test as variables showed normal distribution. A *p* value < 0.05 was considered statistically significant.

## Results

### FXII levels in CDG patients

PMM2-CDG patients showed a median FXII:C of 117% [IQ range 86–152%]. All patients presented FXII values within the normal range, except for 3 cases with lower levels (50–60%). These 3 patients were homozygous TT for the functional *rs1801020* polymorphism.

**Fig. 2:**
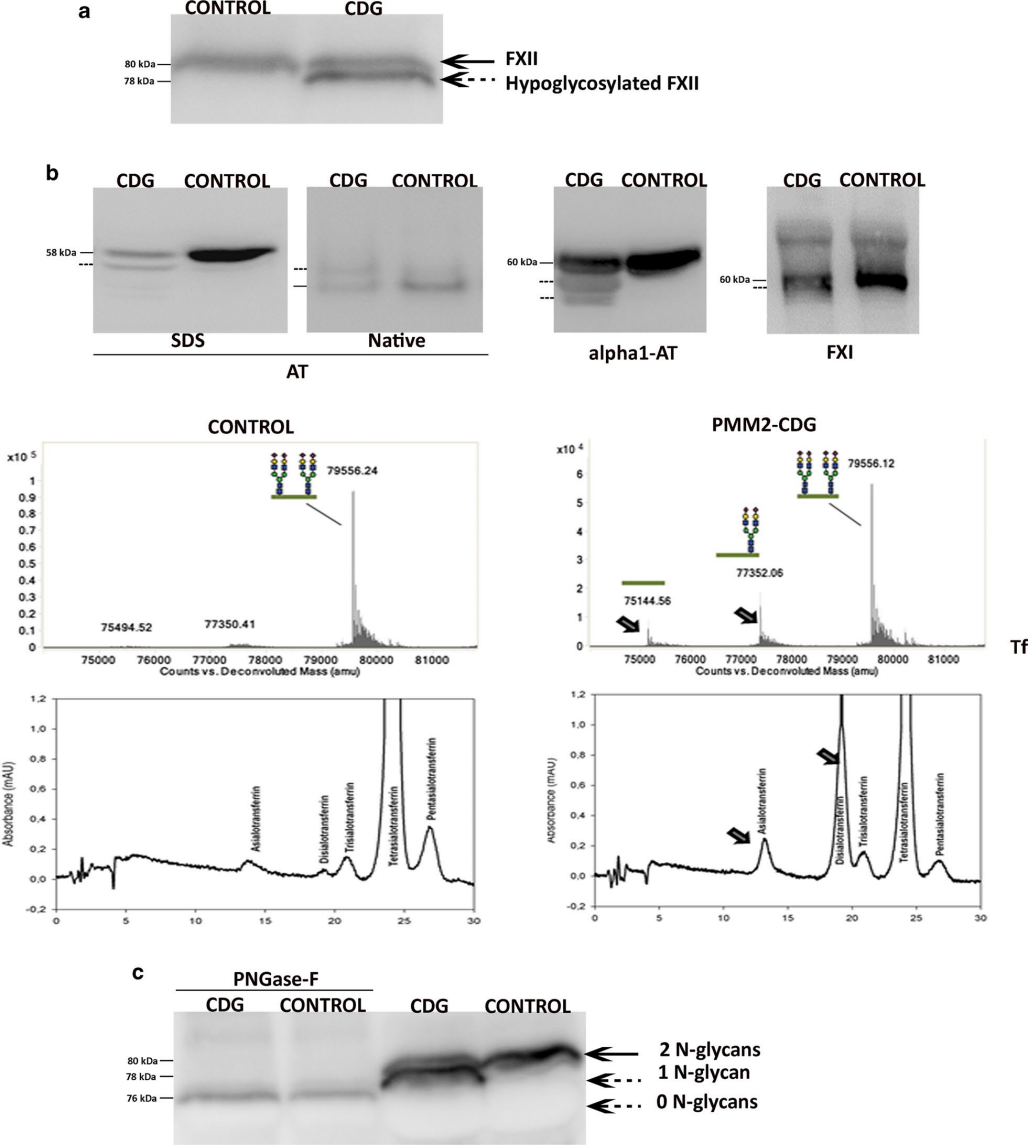
Plasma FXII in a representative PMM2-CDG patient and a healthy subject detected by Western Blot. **a** Basal samples. The aberrant hypoglycosylated form of FXII detected in PMM2-CDG patients (78 kDa) is pointed by a dashed arrow. **b** Identification of increased levels of hypoglycosylated forms of other hepatic proteins (AT: antithrombin; alpha1-AT: α1-antitrypsin; FXI: factor XI; Tf: transferrin) detected by Western Blot, Q-TOF, or HPLC in plasma of the same PMM2-CDG patient. **c** Effect of treatment of plasma with N-glycosidase F (PNGase-F). The FXII forms with 2, 1 and 0 N-glycans (the latter observed after treatment with the enzyme) are marked with arrows

The study of plasma FXII in our cohort of PMM2-CDG patients by Western Blot confirmed that FXII levels in patients were in the normal range and correlated with the *rs1801020* genotype, as carriers of the C/C genotype had almost twice FXII in plasma than carriers of the T/T genotype (Data not shown). However, the most remarkable finding from these studies was the presence of a FXII form with faster electrophoretic mobility in SDS gels in all PMM2-CDG patients. This aberrant form was about 2 kDa smaller than the wild-type according to the data observed in SDS-PAGE under reducing conditions (Fig. 2a). It is important to point out that, as described previously, PMM2-CDG patients had increased levels of hypoglycosylated forms of other hepatic proteins in plasma, such as antithrombin, α1-antitrypsin, FXI or transferrin [19], as verified in our patients by different methods, Western Blot, coagulometric assays, Q-TOF, or HPLC (Fig. 2b).

In order to verify that the aberrant FXII detected in PMM2-CDG is a hypoglycosylated form, plasma of PMM2-CDG and healthy subjects was treated with PNGase-F. This treatment eliminated the heterogeneity of FXII forms in plasma of PMM2-CDG patients, but the resulting FXII without N-glycans (aglyco-FXII) was smaller than the aberrant FXII detected in plasma of patients (Fig. 2c). This result suggested that the hypoglycosylated FXII form detected in plasma of PMM2-CDG patients contains one N-glycan (1 N-glycan-FXII), which is consistent with the size of an N-glycan (2 kDa) and the presence of hypoglycosylated forms of other proteins (antithrombin, FXI, transferrin) in these patients [21, 22]. Moreover, these results also suggested that PMM2-CDG patients had no aglyco-FXII detectable by Western Blot in circulation (Fig. 2c).

**Fig. 3:**
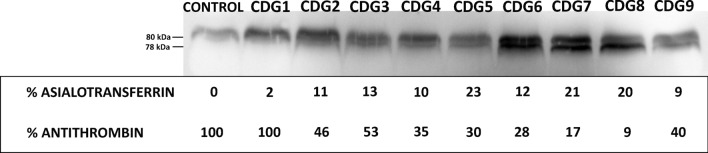
Levels of hypoglycosylated FXII and its relationship with the levels of asialotransferrin (a-Tf) and antithrombin (AT). PMM2 variants in 9 PMM2-CDG patients and a healthy control. Haploins: haploinsuficiency

The levels of the 1 N-glycan-FXII form detected in PMM2-CDG patients were related to the glycosylation defect of each patient, accordingly to the direct correlation observed between the ratio between the two glycoforms determined densitometrically (1 N-glycan/2 N-glycan) and the levels of asialotransferrin (r^2^ = 0.731 and *p* = 0.003). Indeed, an indirect correlation was also observed between this ratio and the anticoagulant activity of antithrombin (r^2^ = 0.456 and *p* = 0.046). Thus, patients with higher asialotransferrin values had lower antithrombin anticoagulant function and high levels of the hypoglycosylated FXII (Fig. 3).

### Clinical characteristics of patients according to FXII

Vascular events were reported in 20 out of these 46 PMM2-CDG patients. Most of these events were stroke-like (n = 12), but also thrombosis (n = 6) and hemorrhages (n = 3) were recorded. No relationship of these vascular events with the levels of FXII were observed (Table 2). However, the presence of vascular events significantly associated with higher levels of hypoglycosylated FXII forms, although this was also true for all markers of hypoglycosylation like asialotransferrin or disialotransferrin, antithrombin or FXI levels (Table 2).

**Table 2:** Levels of FXII and the hypoglycosylated form of FXII as well as other markers of hypoglycosylation in PMM2-CDG patients according to the presence or absence of vascular events

	Vascular events (N = 20)	No vascular complications (N = 26)	*p*
FXII:C (%)	113 ± 48%	120 ± 41%	0.728
1 N/2 N FXII ratio	1.26 ± 0.04	0.54 ± 0.28	*0.003*
Asialo-transferrin (%)	33.5 ± 12.9	21.9 ± 15.1	0.077
Disialo-trasnferrin (%)	12.8 ± 8.4	4.5 ± 5.3	*0.013*
Antithrombin (%)	40.6 ± 23.9	72.0 ± 31.1	*0.001*
FXI (%)	35.6 ± 26.0	74.9 ± 36.30	*0.001*

Statistically significant *p*-values (≤ 0.05) are shown in italics

Finally, no angioedema events were reported in our cohort of PMM2-CDG patients.

### Activation of FXII

**Fig. 4:**
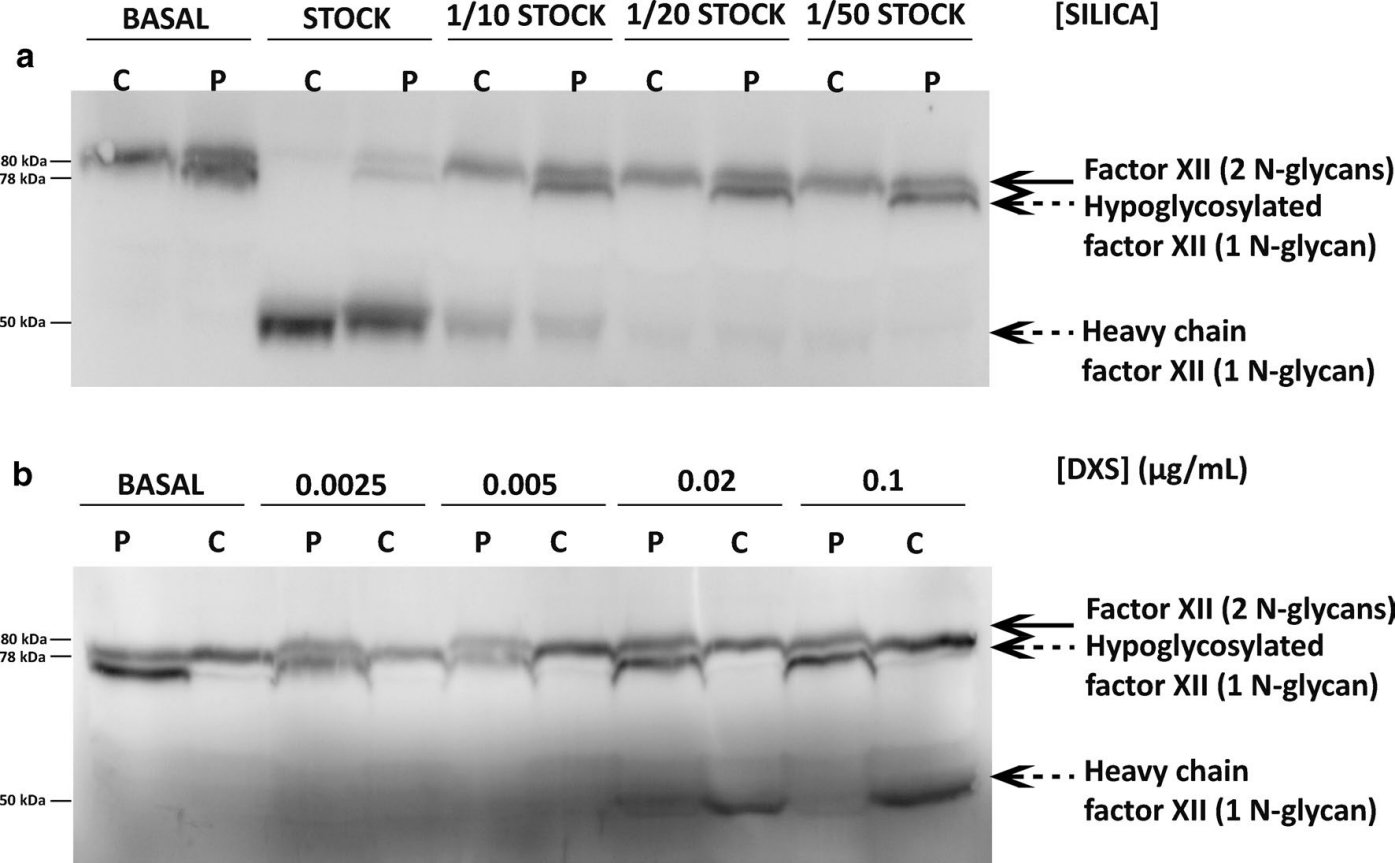
Activation by silica of plasma FXII in a control and a PMM2-CDG patient. The hypoglycosylated form detected at basal conditions in the patient had no equivalence in the heavy chain of the activated form (FXIIa), which was similar to that observed in the control. We used different concentrations of silica (**a**) or dextran sulfate (DXS) (**b**). The inactivated (fully or hypoglycosylated) FXII and the heavy chain detected after activation are pointed by arrows

FXII activation in plasma of PMM2-CDG patients was studied by evaluating the presence of the FXIIa heavy chain by Western Blot, using different concentrations of two activators: silica and DXS. Three interesting results were observed:

Firstly, there was no basal activation of FXII in plasma samples of PMM2-CDG patients (Fig. 4).

Second, after FXII activation the heterogeneity of FXII forms observed in PMM2-CDG patients disappeared, and only a single FXIIa heavy chain was detected, which was similar to that found in healthy controls (Fig. 4). This result means that the hypoglycosylation of FXII in PMM2-CDG patients preferentially affected Asn414, which is located in the light chain. Therefore, most if not all aberrant FXII of PMM2-CDG patients was glycosylated at Asn230 (Fig. 4).

Third, dose-dependent studies using the two activators suggested that the hypoglycosylated form of FXII detected in PMM2-CDG patients was activated in a similar way to the 2-N-glycan form (Fig. 4).

### Recombinant FXII expression

**Fig. 5:**
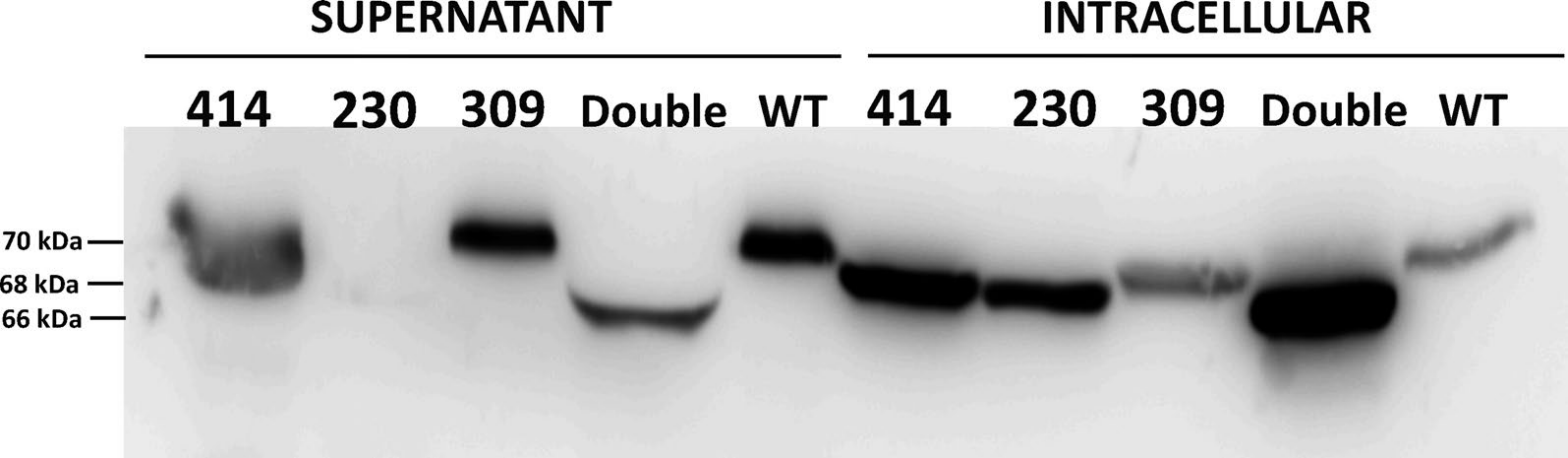
Electrophoretic analysis of the recombinant FXII. Its was secreted into the culture medium (supernatant) and in the cell lysate (intracellular) of Drosophila Schneider 2 cells transfected with the plasmid of wild-type human FXII (WT) or with the following mutations: p.Asn414Lys (414); p.Asn230Lys (230); p.Thr309Lys (309) or the double mutation p.Asn414Lys&p.Asn230Lys (Double)

Analysis of the secreted protein in the culture medium of S2 cells transfected with the plasmids containing the human *F12* cDNA was carried out by Western Blot. Cells transfected with the wild-type plasmid produced high levels of FXII in the culture medium. The secretion and electrophoretic mobility of the Lys309 variant was similar to the wild-type (Fig. 5). In contrast, the expression of the variants affecting N-glycosylation sequons was significantly reduced in the culture medium, especially for the mutant affecting the 230 sequon and the double mutant. Of note, the variant affecting the 414 sequon showed high secretion levels, although they did not reach the levels observed in cells transfected with the wild-type or the Lys309 plasmids (Fig. 5). Furthermore, the variants with a single mutation affecting an N-glycan sequon (230 and 414) showed greater electrophoretic mobility than the wild-type or the Lys309 forms, while the double mutant generated the recombinant FXII with greater electrophoretic mobility in SDS gels, consistent with the loss of one or two N-glycans (Fig. 5).

The study of the intracellular FXII forms of the S2 cells showed that mutants lacking any N-glycosylation and therefore presenting hypoglycosylation (230, 414 and double mutant 230–414) had higher intracellular retention as compared to the wild-type or the p.Lys309 forms (Fig. 5). In addition, the size differences observed in the protein secreted into the medium were maintained in the intracellular protein, again being the double mutant, without any N-glycosylation the form with faster mobility and increased intracellular retention (Fig. 5).

## Discussion

PMM2-CDG is a rare devastating disease affecting mainly children [25]. The defect in the generation of the N-glycan precursor caused by the impaired activity of PMM2 alters the N-glycosylation of potentially all N-glycoproteins. The key role of N-glycosylation for many proteins explains why the resulting hypoglycosylation could affect either the levels or the function of multiple proteins from different organs and tissues [26]. Consequently, the resulting phenotype will have strong biological consequences, particularly during the embryonic development. That explains the severe clinical phenotype of PMM2-CDG patients. It can be associated with multivisceral involvement and also affects the hemostatic system. Thrombosis but also bleeding events, have been described in PMM2-CDG patients as soon as during the perinatal period [25–27]. Our objective was to study the hemostatic system in PMM2-CDG due to the key role that N-glycosylation plays in many different plasma proteins from the hemostatic system [28]. Indeed, several coagulation factors are affected in PMM2-CDG, in particular antithrombin, FXI, protein C, protein S and FIX [27]. In this study, we focused on FXII, an emerging hemostatic molecule that has been involved also in hereditary angioedema. Our data support that FXII levels does not associate to vascular events in these patients, which is in accordance to the low relevance that this factor has for physiological hemostasia both in human patients and animal models [29]. However, we observed an association between these complications and the levels of the hypoglycosylated form of FXII detected in PMM2-CDG patients. This could be explained by the severity of the disease, as hypoglycosylated transferrin and low levels of antithrombin and FXI are also associated to vascular events in our cohort. However, a potential role of this aberrant FXII favouring these events might also be speculated. Indeed, our study aimed to better understand the N-glycosylation of this molecule and its relevance for a correct folding and secretion to the plasma and its activation and role in haemostasis and thrombosis.

The identification of a hypoglycosylated form of FXII in plasma of PMM2-CDG patients, the association of the levels of this aberrant FXII with antithrombin and asialotransferrin levels, and the further analysis of FXII, by using a variety of methods (PNGAse-F treatment, activation by silica or DXS) resulted in the following observations:

First, aglyco-FXII was not detected in plasma of PMM2-CDG patients suggesting that the presence of N-glycosylation is crucial for the folding and secretion of FXII.

Second, our results support that most if not all the hypoglycosylated form of FXII detected in plasma of PMM2-CDG was glycosylated at Asn230, but lacks the N-glycan at Asn414, although further studies including glycoproteomic analysis must be done to demonstrate the aberrant glycosylation of FXII in PMM2-CDG patients. This specific hypoglycosylation might be explained by two mechanisms. (1) The glycosylation of Asn230 is crucial for the correct folding and secretion of FXII, or (2) Asn230 is much more efficiently glycosylated under restrictive concentrations of the glycan precursor. Despite the expression in insect cells have some limitations, such as the different glycan composition than that of mammal cells, the results obtained in the recombinant model support the first hypothesis as the FXII variant altering residue 414 was more efficiently secreted than the 230 variant. Indeed, Asn230 is conserved in FXII across species (83%) (Additional file 1: Fig. 1). Two species (*Usus americanus* and *Mustela putorius furo*) have no Asn at 230 position but instead of this glycosylation site they present an additional N-sequon only 22 residues far from Asn230 (in Asn252), which is also located in the heavy chain (Additional file 1: Fig. 1).

Finally, our study also provides interesting information concerning hereditary angioedema, a disease where FXII plays a significant role. The defective O-glycosylation associated with the *F12* p.Thr309Lys mutation has been involved in an increased sensitivity to the activation of FXII and the development of hereditary angioedema [17]. We might expect that the absence of a complete N-glycan at the C-terminus of FXII might also have the same consequences. However, the data obtained from PMM2-CDG patients do not support an increased activation of the aberrant hypoglycosylated FXII with low doses of the tested activators (silica or DXS) and importantly, no PMM2-CDG patient neither from our cohort nor from the literature has ever developed angioedema to our knowledge. These data support recent studies showing the key role of the N-terminal domain of FXII in the activation of this molecule [30]. However, we must remember that most PMM2-CDG patients have not been exposed to the triggering factors that cause angioedema in carriers of the p.Thr309Lys mutation (pregnancy, oral contraceptives…) [31], and on the other hand that marked edemas were already reported in CDG patients [32–36].

To sum up, our study has revealed new information on FXII, one emerging molecule in different disorders, namely the critical role that glycosylation at Asn230 has in the efficient secretion of FXII to the plasma. Moreover, our study opens new perspectives on the relevance that glycosylation might have in the contact pathway. Finally, CDG patients undoubtedly are an excellent source of information to understand the relevance of N-glycosylation for other glycoproteins and disorders. However, we also must take into account that, as any other complex disease, CDG may be accompanied by compensatory mechanisms that might affect N-linked glycan synthesis or the functional consequences of one aberrant molecule.

## Conclusions

In this study, we characterized the glycosylation of FXII, the first element of the contact pathway in PMM2-CDG patients. These patients present high levels of a homogeneous FXII hypoglycosylated form without N-glycosylation at Asn414. This, and the results obtained in the recombinant model support that N-glycosylation at Asn230 is essencial for the correct folding and/or secretion of FXII. Finally, the results confirm that this glycoform is similarly activated than fully glycosylated FXII, explaining the absence of angioedema in PMM2-CDG patients.

## Supplementary information

Additional file 1: Fig. 1. Conservation of N-glycosylation sequons in FXII from 12 species.

## Data Availability

Not applicable.

## Abbreviations

CDG: Congenital disorders of glycosylation
PPM2: Phosphomannomutase 2
FXII: Factor XII
HAE: Hereditary angioedema
FXI: Factor XI
BK: Bradykinin
FXIIa: Activated FXII
PTM: Post-translational modification
PAGE: Polyacrilamide electrophoresis
DTT: Dithiothreitol
PNGaseF: N-glycosidase F
DXS: Dextran sulfate
Asp: Asparagine
Lys: Lysine
Thr: Threonine
SFM: Serum-free Express Five
