# Supplementary figures and images for: Factor XII in PMM2-CDG patients: role of N-glycosylation in the secretion and function of the first element of the contact pathway
Source: Orphanet J Rare Dis. 2020 Oct 9;15:280. doi: 10.1186/s13023-020-01564-9 (PMC7547467; doi:10.1186/s13023-020-01564-9)

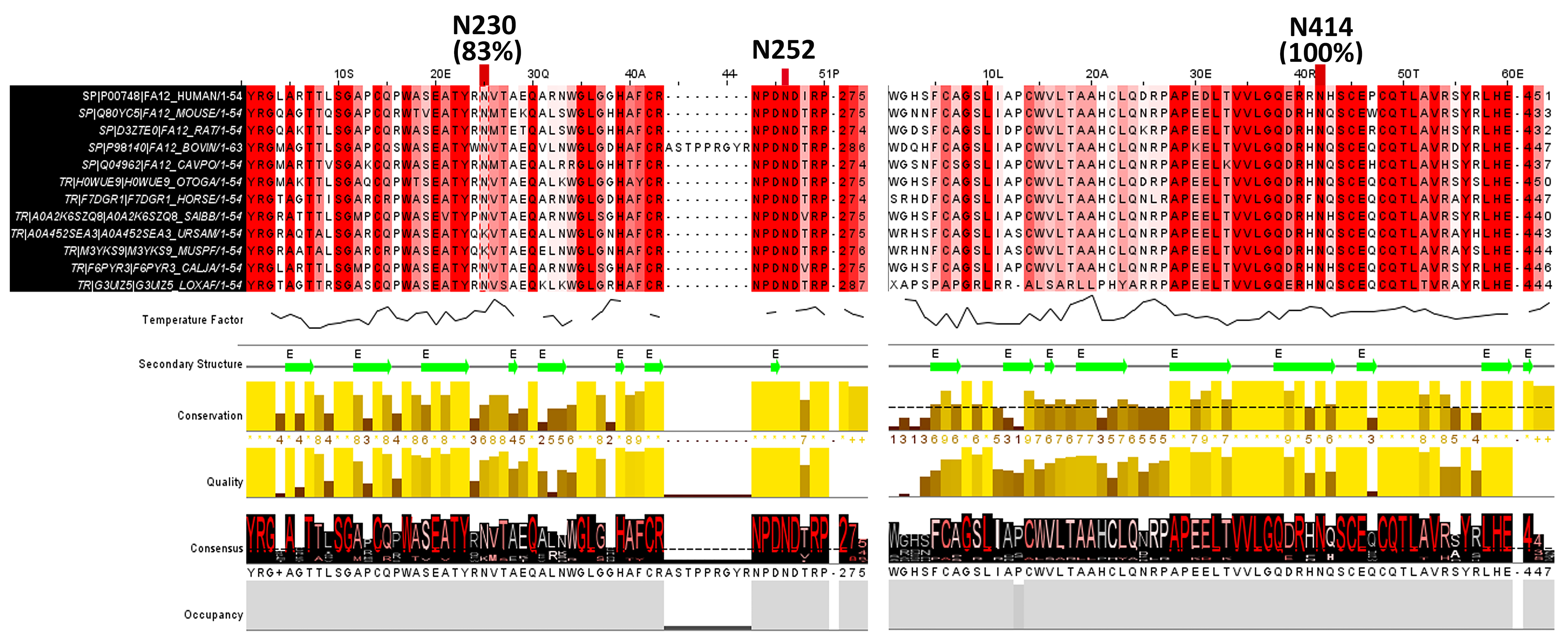

Supplement: Supplementary file 1 — Conservation of N-glycosylation sequons in FXII from 12 species. [file 13023_2020_1564_MOESM1_ESM.tif]
